# Minor allele of rs55763075 located in MTHFR is associated with the risk of cognitive impairment after anesthesia via modulating miR-34b

**DOI:** 10.1038/s41598-021-90229-z

**Published:** 2021-05-27

**Authors:** Si-ying Li, He-shou Lei, Xiao-yun Wu, Kai Li, Zhi-min Liu, Jian-hui Lu, Xiao-yun Chen

**Affiliations:** Department of Anesthesiology, Wuming Hospital of Guangxi Medical University, No.26 Yongning Road, Wuming District, Nanning, 530199 Guangxi Zhuang Autonomous Region People’s Republic of China

**Keywords:** Cell biology, Genetics

## Abstract

This study aimed to investigate the association between cognitive impairment after general anesthesia and rs55763075 polymorphisms. We enrolled and grouped patients undergoing general anesthesia according to their genotypes of rs55763075 polymorphism. Mini–Mental State Examination (MMSE) scoring was performed to evaluate the cognitive status of patients. Quantitative real-time PCR was carried out to analyze the expression of methylenetetrahydrofolate reductase (MTHFR) mRNA and miR-34b while Western blot was performed to evaluate the expression of MTHFR protein. Furthermore, we studied the effect of rs55763075 polymorphism on the expression of MEHFR via luciferase assay. Accordingly, we found that the MMSE score in GG/GA groups was significantly higher than that in AA group. And a significant reduction of MTHFR mRNA expression was observed in the serum and peripheral blood mononuclear cells (PBMCs) of patients carrying AA genotype compared with the patients carrying GG/GA genotypes. Moreover, the MTHFR expression was much lower in the cultured AA-genotyped cells transfected with miR-34b. Luciferase assay results also showed that miR-34b transfection reduced luciferase activity in the cells carrying A allele but not in cells carrying G allele. In summary, the data of this study showed that minor allele (A) of rs55763075 polymorphisms in the 3'-untranslated region of MTHFR mRNA generated a potential binding site for miR-34b, which led to reduced level of folic acid in the patients carrying the AA genotype. Furthermore, we found that the MMSE score of AA-genotyped patients was lower than that of patients carrying GG/GA genotypes.

## Introduction

The term of postoperative cognitive dysfunction (POCD) is specified as cognitive downtrend recognized by utilizing certain techniques. The field of POCD research determined that minor cognitive downtrend in elderly people can be observed in the postoperative period although many might not show evident signs unless evaluated through neuropsychological screening. It is currently recognize that minor cognitive decrease can develop in elderly people. The cognitive downtrend shown in patients undergoing anesthesia has drawn great attention.

Folate, also called vitamin B9, is the universal term for a group of chemically comparable substances that have been identified as beneficial for avoiding a variety of disorders. Folate is a crucial trace element that is important for typical cell functionality. An enough level of folate uptake is an important factor in protecting against neural tube defects. Folate has been proved to be associated with several damaging ailments including anemia, heart diseases and cancers^1–3^. Plasma content of folate is negatively associated with concentrations of homocysteine (Hcy) in the blood^4,5^. Folic acid on its own possesses no coenzyme properties^6^. However, tetrahydrofolate (THF) is metabolized by serine hydroxy methyltransferase (SHMT) to produce glycine as well as 5,10-methylene-THF, which in turn converts to L-5-methyl-THF in blood due to the activity of MTHFR^1,7^. MTHFR is the principal chemical involved in the metabolic process of folic acid to transform 5,10-methylenetetrahydrofolate to 5-methyltetrahydrofolate, an important substance required for remethylating of Hcy to methionine^8,9^. The 5-methyltetrahydrofolate-homocysteine methyltransferase (MTR) gene encodes methionine synthase to induce the transfer from 5-methyltetrahydrofolate to Hcy and created methionine, which is subsequently converted into S-adenosylmethionine to contribute a methyl group for DNA methyltransferases^10^. The hypomethylation of DNA was shown to induce the A2756G polymorphism in the MTR gene^8,11^.

MicroRNA (miRNA) is a type of noncoding RNA which moderates the expression of certain mRNAs by binding to these mRNAs in the 3'-UTR^12^. And the single nucleotide polymorphism (SNP) in miRNA binding locations has been reported to possibly change the level of miRNA expression^13^. It was also discovered that miR-34b hindered osteoblast expansion in mice through targeting Satb2 Bae presented that miR-34b/c participated in bone tissue homeostasis, partly by regulating Notch signaling^14,15^. Moreover, a SNP named rs55763075 was found in the miR-34b binding site on MTHFR gene. More evidence has proposed that miR-34b is associated with several cellular processes including spermatogenesis^16,17^. When compared to homozygous carriers of rs55763075 GG, the carriers of MTHFR rs55763075 AA showed a statistically higher risk of infertility. The genotype of rs55763075 AA was linked to reduced folate content as well as boosted level of Hcy in individuals with azoospermia^18^.

It has been shown that folic acid level is associated with the risk of cognitive impairment in the subjects receiving general anesthesia, and the minor allele of rs55763075 polymorphism in the 3’-UTR of MTHFR generates a binding site of miR-34b, leading to reduced expression level of MTHFR^18,19^. In this study, we enrolled subjects undergoing general anesthesia and investigated the association between rs55763075 polymorphism and cognitive status.

## Materials and methods

### Human subjects and sample collection

We recruited 193 patients who had received general anesthesia. Then, we performed genotyping on rs55763075 of all participants. These patients were divided into three groups according to their genotypes at rs55763075: 1) GG (N = 79), 2) GA (N = 89), 3) AA (N = 25). The information of participants including their age, weight, sex, length of anesthesia and past medical history was collected and compared among the three groups. This study was approved by the Ethical committee of Wuming Hospital of Guangxi Medical University and was performed in strict accordance with the last vision of the Declaration of Helsinki. Written informed consent was obtained from all patients or their first-degree relatives before the study.

### Evaluation of post-operative cognitive status by MMSE scoring

Since the single nucleotide polymorphism in the ApoE gene differs in people of different ethnic backgrounds, we only enrolled Chinese Han subjects who have undergone general anesthesia at our hospital. All subjects were arbitrarily assigned in different orders to receive general anesthesia at different points. During participant screening, patients who were subjected to postoperative treatment due to inflammation, blood loss, cardiac arrest, respiratory failure, or anastomotic leaks were excluded. All enrolled participants had a score of American Society of Anesthesiologists Physical Status of I to II. The mean age of the enrolled participants was 70.1 ± 4.6 years old, while the body weight of the enrolled participants was 57.3 ± 7.5 kg. Senior participants with dementia symptoms were excluded based on the 3rd edition of the American Psychiatric Disease Diagnosis and Statistics Handbook and Folstein MMSE standard. Based on the above standards, the participants with a MMSE score of at least 25 points had little cognitive problems prior to anesthesia.

### RNA isolation and real-time PCR

Serum and peripheral blood samples were collected from all participants. PBMCs were then isolated from collected peripheral blood samples. To measure the expression of MTHFR mRNA and miR-34b in each sample, the samples were first homogenized and treated with a Trizol reagent kit (Invitrogen, Carlsbad, CA) according to the manufacturer instruction. For qualitative examination of RNA integrity, 2 μg of isolated total RNA in each sample were electrophoresed by using a 1% agarose gel, and only qualified RNA samples were used for subsequent analyses. For assessment of MTHFR mRNA and miR-34b expression, 1 μg of isolated total RNA in each sample was reverse transcribed by making use of an iScript reverse transcriptase assay kit (Bio Rad Laboratories, Hercules, CA) according to the procedures provided by the manufacturer on the instruction manual of the experimental kit, while the miRNA content in samples was directly evaluated by making use of a Taqman real time RT-PCR assay kit (Invitrogen, Carlsbad, CA) according to the procedures provided by the manufacturer on the instruction manual of the experimental kit. In addition, the synthesized cDNA was subjected to real time PCR by using a Fast EvaGreen Supermix kit (Bio Rad Laboratories, Hercules, CA) according to the procedures provided by the manufacturer on the instruction manual of the experimental kit. The relative the expression of MTHFR mRNA and miR-34b in each sample was calculated by using the Ct method, and GAPDH and Let-7a was respectively used as the internal control for the calculation of relative expression of MTHFR mRNA and miR-34b.

### Cell culture and transfection

Isolated PBMCs were grown in T175 Corning flasks (Corning, Corning, NY) in DMEM (Gibco, Thermo Fisher Scientific, Waltham, MA) added with 10% heat inactivated fetal bovine serum (FBS, Gibco, Thermo Fisher Scientific, Waltham, MA) and appropriate antibiotics. The culture was carried out at 37 °C and 5% carbon dioxide in a humidified tissue culture incubator. Then, the cells carrying the AA genotype of rs55763075 SNP were divided into 3 groups, i.e., 1. NC group (cells transfected with a negative control); 2. miR-34b precursor group (cells transfected with miR-34b precursors); and 3. MTHFR siRNA group (cells transfected with MTHFR siRNA). Similarly, the cells carrying the GG genotype of rs55763075 SNP were also divided into 3 groups, i.e., 1. NC group (cells transfected with a negative control); 2. miR-34b precursor group (cells transfected with miR-34b precursors); and 3. MTHFR siRNA group (cells transfected with MTHFR siRNA). The siRNAs used in this study were generated by Qiagen (Hilden, Germany). Cell transfection was carried out by using Lipofectamine 2000 (Invitrogen, Carlsbad, CA) according to the procedures provided by the manufacturer on the instruction manual of the experimental kit. The transfected cells were harvested at 48 h post transfection to assay the expression of target genes.

### Cell proliferation assay

The proliferation of cell samples was evaluated by using an MTT assay kit (Sigma Aldrich, St. Louis, MO) according to the instructions. The absorbance of each well was measured at 490 nm wave length by using a Modulus ELISA reader (Turner Bio Systems, Sunnyvale, CA).

### Vector construction, mutagenesis and luciferase assay

To explore the effect of rs55763075 SNP on the binding capability of miR-34b to MTHFR, we constructed luciferase plasmids in which the a specific site on the sequence of rs55763075 SNP was changed from G to A by a site directed mutagenesis assay kit (Stratagene, San Diego, CA) according to the manufacturer instructions. Subsequntly, THP-1 and Hep G2 cells were divided into 3 groups, i.e., 1. NC group (cells transfected with a negative control); 2. miR-34b + MTHFR-3’UTR-A group (cells transfected with miR-34b mimics and MTHFR-3’UTR-A); 3. miR-34b + MTHFR-3’UTR-G group (cells transfected with miR-34b mimics and MTHFR-3’UTR-G). Transfection was accomplished using Lipofectamine 2000 and the luciferase activity of transfected cells was assayed 48 h late by a Dual Luciferase Assay kit (Promega, Madison, WI) according to the the manufacturer instructions.

### Western blot analysis

Protein was isolated from samples by using a RIPA reagent kit (Sigma Aldrich, St. Louis, MO) according to the procedures provided by the manufacturer on the instruction manual of the experimental kit. Then, the concentration of isolated proteins was measured by using a BCA assay kit (Beyotime, Jiangsu, China) according to the procedures provided by the manufacturer on the instruction manual of the experimental kit. In the next step, equal quantity of protein was resolved by using SDS-PAGE and blotted onto an Immobilon P membrane, which was then blocked with 5% skim milk and incubated with anti-MTHFR primary antibodies and suitable secondary antibodies (Abcam, Cambridge, MA) consecutively. After being developed with ECL reagents, the relative protein expression of MTHFR in each sample was evaluated by making use of Quantity One 4.4 (Bio Rad Laboratories, Hercules, CA) software.

### Statistical analysis

Results shown in here were all derived from at least 3 independent experiments. The data were shown as mean ± SD. Statistical analysis was done by using SPSS software (SPSS, Chicago, IL). Chi square test was utilized to perform the statistical comparison upon the non-continuous variables in Table 1. And student’s t-test was performed to compare the difference among two different groups, while one-way ANOVA was performed to compare the differences among multiple groups. P < 0.05 was considered significant.

**Table 1:** Basic information of participants recruited in this study.

Characteristics	GG (N = 79)	GA (N = 89)	AA (N = 25)	*P* value
Age, year	69.54 ± 5.62	68.94 ± 5.75	71.54 ± 4.85	0.734
Weight (kg)	58.82 ± 6.64	59.45 ± 7.78	58.54 ± 8.45	0.543
Sex, male	35 (44.3)	45 (50.6)	17 (68.0)	0.242
Length of anesthesia (h)	3.09 ± 0.29	3.16 ± 0.14	3.09 ± 0.28	0.622
Past medical history	No serious heart lung diseases. Hepatic or renal dysfunction			

## Results

### The characteristics of patients

We recruited 193 patients who were subjected to general anesthesia and grouped these patients according to their genotypes at rs55763075 as GG group, GA group and AA group. The information of participants including their age, weight, sex, length of anesthesia and past medical history was collected and listed in Table 1. And the statistical analysis revealed that there was no obvious difference in all above characters between the three groups.

### The MMSE score was remarkably decreased for patients with AA genotype at rs55763075

**Figure 1:**
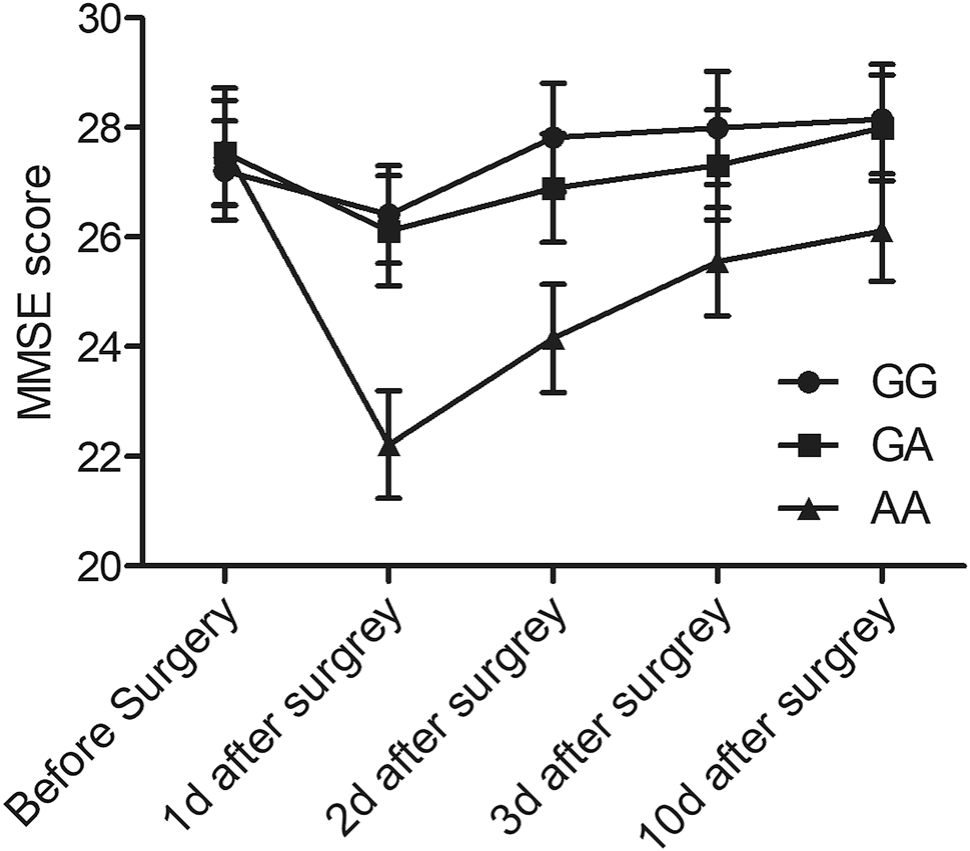
The MMSE score was significantly lower for patients carrying AA alleles.

MMSE scoring was carried out to evaluate the cognitive status of the patients in the three groups. As shown in Fig. 1, no obvious difference was observed for the MMSE scores between patients with GG and GA genotypes, whereas the MMSE score was significantly deceased for patients with AA genotype.

### The concentration of folic acid and Hcy in the peripheral blood of patients with AA genotype was obviously altered

**Figure 2:**
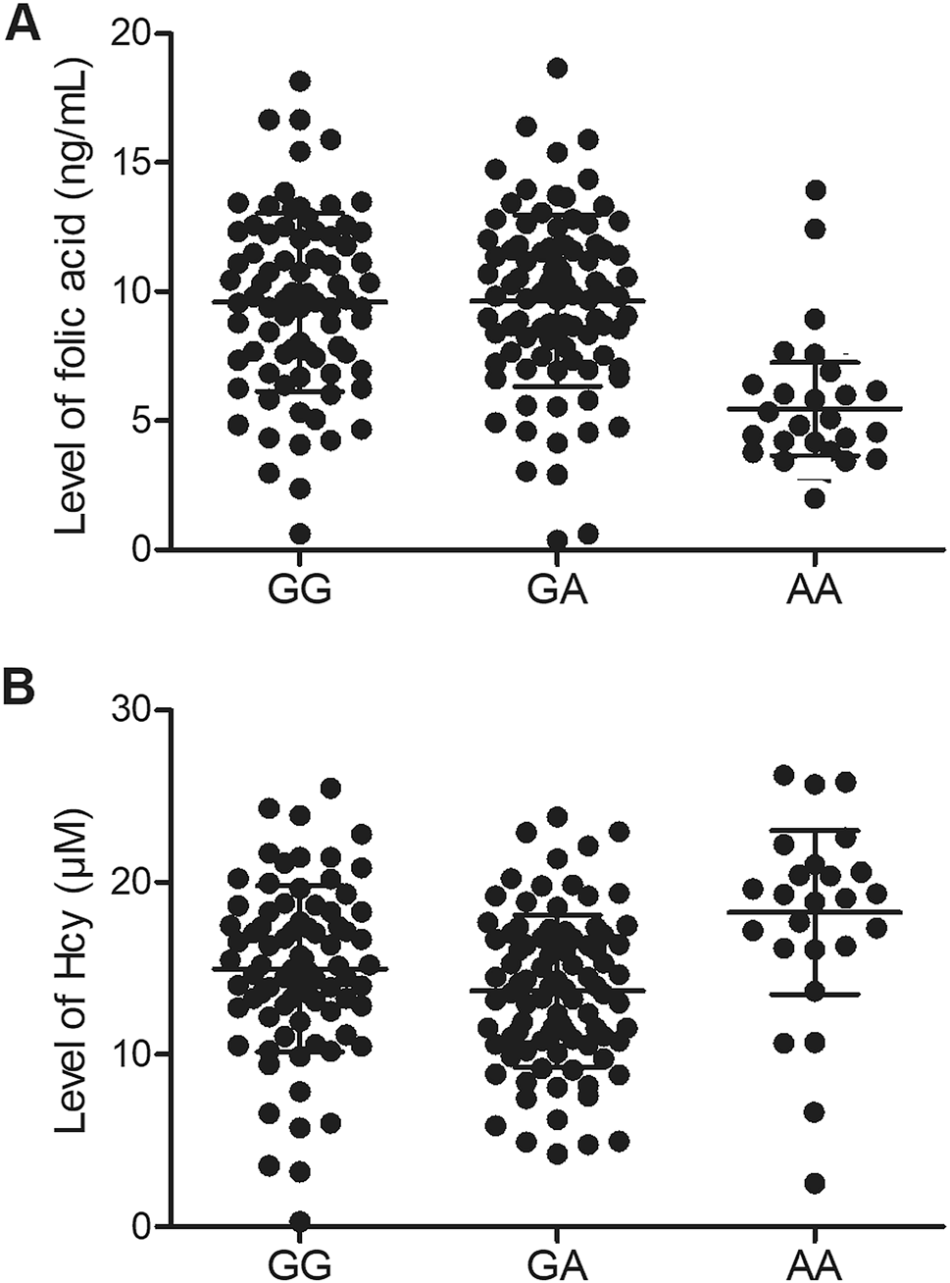
Polymorphism at rs55763075 was correlated with folic acid and Hcy concentrations in the peripheral blood of patients who had received general anesthesia. (**A**) The concentration of folic acid was lower in the peripheral blood of patients carrying AA alleles. (**B**) The concentration of Hcy was higher in the peripheral blood of patients carrying AA alleles.

As the host gene of rs55763075 was proven to be directly involved in the folic acid metabolism, we further analyzed the concentration of folic acid and Hcy in the peripheral blood of patients carrying different genotypes of rs55763075. The concentration of folic acid was notably diminished in the peripheral blood of patients carrying AA alleles (Fig. 2A). On the contrary, the level of Hcy in the peripheral blood of patients carrying AA alleles was apparently elevated (Fig. 2B).

### The expression of MTHFR mRNA was notably suppressed in the serum and PBMCs of patients carrying AA genotype of rs55763075

**Figure 3:**
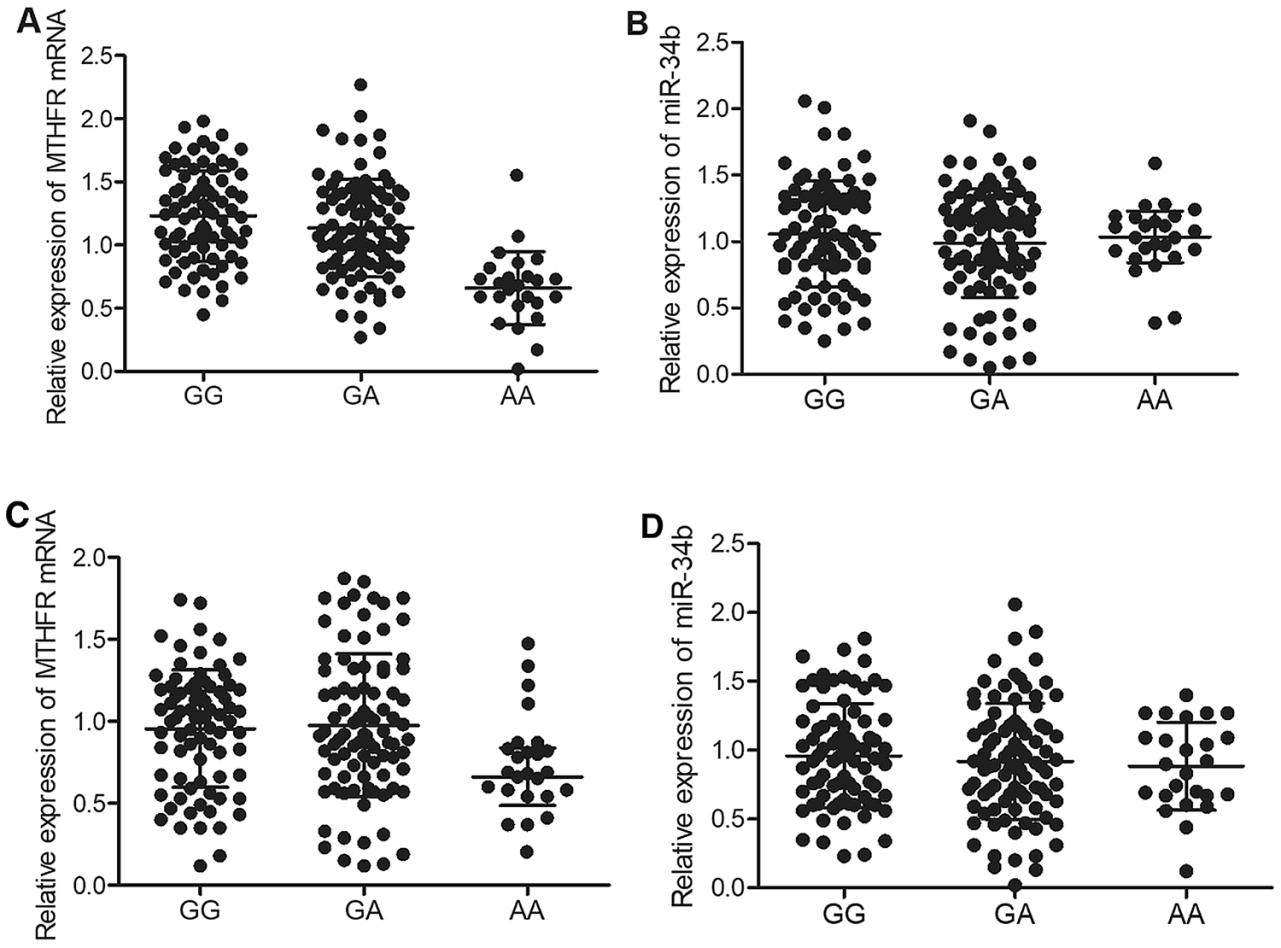
The expression of MTHFR mRNA was decreased in the serum and PBMCs of patients carrying AA alleles of rs55763075. (**A**) MTHFR mRNA expression was notably diminished in the serum of patients carrying AA alleles. (**B**) No obvious difference was observed for the expression of miR-34b in the serum of patients carrying different genotypes. (**C**) MTHFR mRNA expression was notably diminished in the PBMCs of patients carrying AA alleles. (**D**) No obvious difference was observed for the expression of miR-34b in the PBMCs of patients carrying different genotypes.

Furthermore, according to the results obtained from quantitative real-time PCR upon the expression of MTHFR mRNA and miR-34b in the serum and PBMCs of patients carrying different genotypes, it was demonstrated that the expression of MTHFR in the serum (Fig. 3A) and PBMCs (Fig. 3C) of patients carrying AA alleles was significantly suppressed. However, no obvious difference was observed for the expression of miR-34b in the serum (Fig. 3B) and PBMCs (Fig. 3D) of patients carrying GG, GA and AA genotypes.

### The luciferase activity of MTHFR plasmid containing AA genotype of rs55763075 was remarkably suppressed by miR-34b

**Figure 4:**
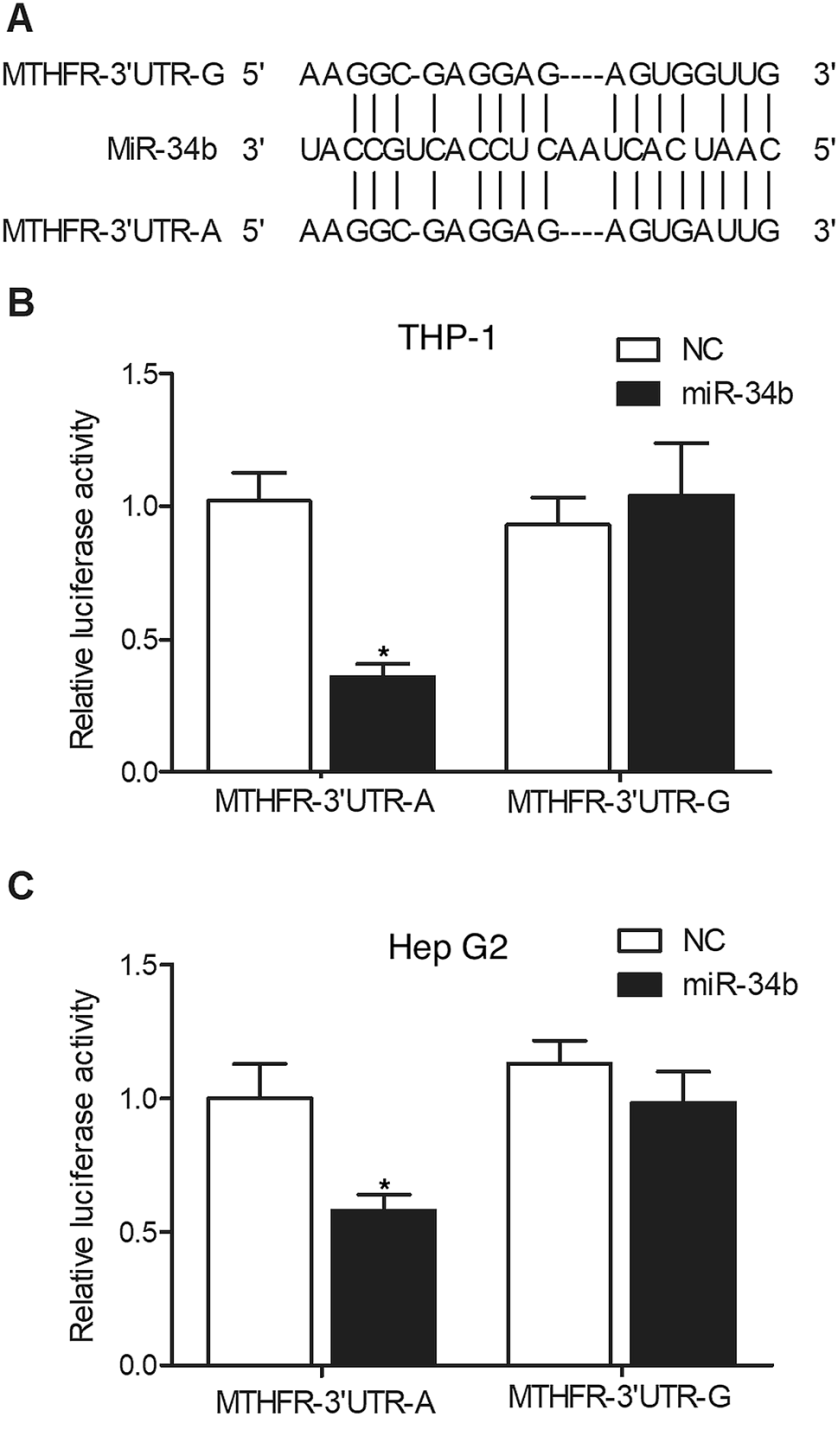
MiR-34b inhibited the luciferase activity of MTHFR-3’UTR-A (* P value < 0.05 vs. NC group). (**A**) Sequence analysis indicated potential binding of miR-34b to the 3’ UTR of MTHFR containing rs55763075. (**B**) The luciferase activity of MTHFR-3’UTR-A was effectively suppressed by miR-34b in THP-1 cells. (**C**) The luciferase activity of MTHFR-3’UTR-A was effectively suppressed by miR-34b in Hep G2 cells.

We further performed sequence analysis and found that the 3’ UTR of MTHFR contains a candidate binding site for miR-34b (Fig. 4A). To explore the effect of rs55763075 on miR-34b binding capability, luciferase plasmids containing MTHFR-3’UTR-A and MTHFR-3’UTR-G were constructed and transfected into THP-1 cells with miR-34b. The luciferase activity of MTHFR-3’UTR-A vector was obviously repressed by miR-34b in THP-1 cells, while no inhibition was observed for MTHFR-3’UTR-G (Fig. 4B). Moreover, these results were further validated in Hep G2 cells (Fig. 4C). Therefore, it can be proved that rs55763075 influenced the binding capacity of miR-34b on its target gene MTHFR.

### MiR-34b suppressed the expression of MTHFR in primary PBMCs of patients carrying AA genotype at rs55763075

**Figure 5:**
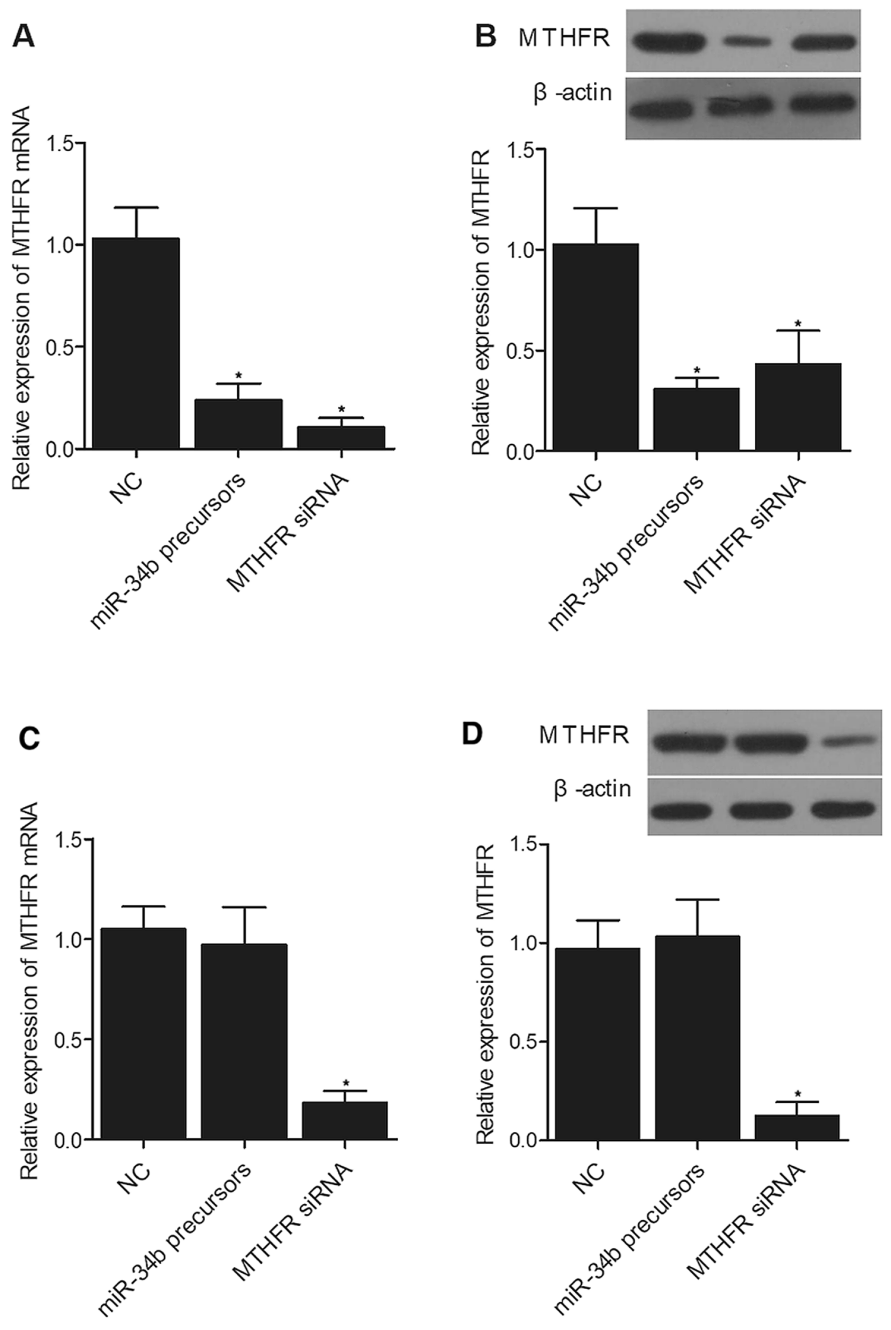
MiR-34b precursors suppressed the expression of MTHFR mRNA and protein in the primary PBMCs of patients carrying AA genotype of rs55763705 rather than in the primary PBMCs of patients carrying GG genotype of rs55763705 (*P value < 0.05 vs. NC group). (**A**) The expression of MTHFR mRNA was inhibited by miR-34b precursors and MTHFR siRNA in primary PBMCs of patients carrying AA genotype of rs55763705. (**B**) The expression of MTHFR protein was inhibited by miR-34b precursors and MTHFR siRNA in primary PBMCs of patients carrying AA genotype of rs55763705. (**C**) The expression of MTHFR mRNA was not affected by miR-34b precursors in primary PBMCs of patients carrying GG genotype of rs55763705. (**D**): The expression of MTHFR protein was not affected by miR-34b precursors in primary PBMCs of patients carrying GG genotype of rs55763705. (the original WB blots were included in Supplementary files)

In order to gain a deep insight into the regulatory network involving miR-34b and rs55763075 polymorphism, we isolated primary PBMCs from patients carrying AA and GG alleles at rs55763075. MiR-34b precursors and MTHFR siRNA were transfected into primary PBMCs carrying different genotypes, and the expression of MTHFR was analyzed using qPCR and Western blot. The expression of MTHFR mRNA (Fig. 5A) and protein (Fig. 5B) was obviously suppressed by miR-34b precursors in primary PBMCs carrying AA alleles at rs55763075. However, the expression of MTHFR mRNA (Fig. 5C) and protein (Fig. 5D) in primary PBMCs carrying GG alleles at rs55763075 remained unchanged when transfected with miR-34b precursors, although MTHFR siRNA also effectively suppressed the expression of MTHFR mRNA and protein in primary PBMCs carrying GG alleles. These results indicated that AA genotype facilitated the binding of miR-34b to the 3’ UTR of MTHFR, and miR-34b effectively repressed the expression of MTHFR in patients carrying AA genotype.

## Discussion

There have been numerous studies investigating the relationship between cognitive functions and anesthesia as well as surgical procedures^20^. Numerous pathways are involved in the pathogenesis underlying cognitive conditions and neurodegeneration diseases induced by anesthesia, such as oxidative stress, toxicity mediated by N-methyl-D-aspartate, reductions in transduction of cholinergic signaling, as well as augmentation of protein oligomerization^21^. Gene polymorphisms in the gene of apolipoprotein E (ApoE) has been reported to be linked to senile dementia^22–24^. The ApoE gene has 3 allelic genes, including ε3, ε4, as well as ε2, which may generate 6 genotypes of homozygotes as well as heterozygotes, which are then transformed to 6 phenotypes. In senile individuals, the frequency of the ApoE3 gene is highest, hence the ε3/ε3 genotype is most usual^25^. In this study, we recruited 193 patients who had received general anesthesia and explored the effect of rs55763075 polymorphisms on the risk of cognitive impairment. The MMSE score of patients carrying AA genotype of rs55763075 was significantly lower than that in patients carrying GG and GA genotypes. If a variant affects a phenotype, it either directly or indirectly affects the expression of a functionally related gene.

Folate, also referred to as vitamin B9, is a necessary component crucial for maintaining typical cell functions. Inadequate intake of folate has been linked to the onset of nerve organs cylinder issues. Folate has also been linked to some types of anemia as well as heart diseases or cancer^1,26^. A link between folate consumption and decreased risk of cognitive disorders has also been speculated^4,5^. It has been revealed that B vitamin therapies can provide statistically considerable benefit to cognitive functions. It was discovered that higher dosages of B vitamins can help cognitive functions^27,28^. Moreover, patients that suffer from memory disorders or even cognitive problems might forget to consume food, which also impacts their vitamin intake. Furthermore, memory disorders can cause people to forget brushing teeth, causing dental health issues^29,30^. MTHFR is a critical catalytic enzyme that is functionally involved in the metabolic process of folic acid to transform 5,10-methylenetetrahydrofolate to 5-methyltetrahydrofolate, an important substance required for remethylation of Hcy to methionine^8,9^. In this study, we analyzed the concentration of folic acid and Hcy in the peripheral blood of patients carrying different genotypes of rs55763075. The concentration of folic acid was notably decreased in the peripheral blood of patients carrying AA genotype. The concentration of Hcy was remarkably increased in the peripheral blood of patients carrying AA genotype. In addition, we performed qPCR to assess the expression of MTHFR mRNA and miR-34b in the serum and PBMCs collected from patients carrying different genotypes of rs55763075. The expression of MTHFR mRNA was apparently decreased in the serum and PBMCs of patients carrying AA alleles, whereas no obvious difference was observed for miR-34b in different groups. This findings are in line with our hypothesis that rs55763075 polymorphism located in the 3’UTR MTHFR may interfere with the interaction between miRNA and the target gene leading to dysregulation of MTHFR as well as dysmetabolism of folic acid which might the molecular mechanism underlying anesthesia associated cognitive impairment.

It has been revealed that the rs55763075 SNP located in the 3' UTR of MTHFR gene responsible for miR-34b binding site can affect male productivity^18^. It was shown that the rs55763075 SNP is linked with the onset of idiopathic azoospermia^18^. In this study, we performed luciferase assay to explore the regulatory role of miR-34b in the expression of MTHFR. The expression of MTHFR-3UTR-A was remarkably suppressed by miR-34b, but no inhibition was observed for MTHFR-3’UTR-G. MTHFR is an essential enzyme that can catalyze the transformation of 5,10-methylenetetrahydrofolate to 5-methyltetra-hydrofolate^31^. Latest study has highlighted the role of MTHFR in male productivity. A research on mice showed that MTHFR participates in an essential process of spermatogenesis^32^. Mice lacking MTHFR also display hyperhomocysteinemia as well as disorders in spermatogenesis^33^. Typical variants of MTHFR are linked to reduced enzyme activity, reduced level of folate in blood, as well as high plasma level of Hcy^34^. Many researchers have discovered that MTHFR polymorphisms are related to changed MTHFR enzyme activities as well as hyperhomocysteinemia^35^. It was additionally presented that the genotypes of MTHFR participate in the change in the state of mind as well as cognitive functions. On top of that, cognitive scores do not seem to be connected with levels of Hcy, folate or B12^36–38^.

## Conclusion

The findings of this study demonstrated that minor allele (A) of rs55763075 polymorphisms in the 3'-untranslated region of methylenetetrahydrofolate generated a potential binding site of miR-34b, leading to reduced level of folic acid in the subjects carrying AA genotype. Furthermore, we found that the MMSE score in AA group was lower than that of G groups.

## Supplementary Information

Supplementary Information.

## Data Availability

The data that support the findings of this study are available from the corresponding author upon reasonable request.

## References

[1] Blom HJ, Smulders Y (2011). Overview of homocysteine and folate metabolism. With special references to cardiovascular disease and neural tube defects. J. Inherit. Metab. Dis..

[2] Czeizel AE, Dudas I (1992). Prevention of the first occurrence of neural-tube defects by periconceptional vitamin supplementation. N. Engl. J. Med..

[3] Klerk M (2002). MTHFR 677C–>T polymorphism and risk of coronary heart disease: a meta-analysis. JAMA.

[4] Forman JP (2005). Folate intake and the risk of incident hypertension among US women. JAMA.

[5] Jardine MJ (2012). The effect of folic acid based homocysteine lowering on cardiovascular events in people with kidney disease: Systematic review and meta-analysis. BMJ.

[6] Luo M (2018). Enhanced stability and oral bioavailability of folic acid-dextran-coenzyme Q10 nanopreparation by high-pressure homogenization. J. Agric. Food Chem..

[7] Gregory JF (2000). Primed, constant infusion with [2H3]serine allows in vivo kinetic measurement of serine turnover, homocysteine remethylation, and transsulfuration processes in human one-carbon metabolism. Am. J. Clin. Nutr..

[8] Salimi S (2017). Polymorphisms of the folate metabolizing enzymes: Association with SLE susceptibility and in silico analysis. Gene.

[9] Wang X (2016). Geographical and ethnic distributions of the MTHFR C677T, A1298C and MTRR A66G gene polymorphisms in Chinese populations: A meta-analysis. PLoS ONE.

[10] Belfeki N (2018). Thrombophilia in systemic lupus erythematosus: A case-control study. J. Med. Vasc..

[11] Burzynski M (2007). MTR 2756 A > G polymorphism is associated with the risk of systemic lupus erythematosus in the Polish population. Lupus.

[12] Bartel DP (2004). MicroRNAs: Genomics, biogenesis, mechanism, and function. Cell.

[13] Sethupathy P, Collins FS (2008). MicroRNA target site polymorphisms and human disease. Trends Genet..

[14] Wei J (2012). miR-34s inhibit osteoblast proliferation and differentiation in the mouse by targeting SATB2. J. Cell. Biol..

[15] Choe N (2015). The microRNA miR-34c inhibits vascular smooth muscle cell proliferation and neointimal hyperplasia by targeting stem cell factor. Cell Signal.

[16] Abu-Halima M (2014). MicroRNA expression profiles in human testicular tissues of infertile men with different histopathologic patterns. Fertil. Steril..

[17] Abu-Halima M (2013). Altered microRNA expression profiles of human spermatozoa in patients with different spermatogenic impairments. Fertil. Steril..

[18] Zhang W (2015). Association of a miR-34b binding site single nucleotide polymorphism in the 3'-untranslated region of the methylenetetrahydrofolate reductase gene with susceptibility to male infertility. Genet. Mol. Res..

[19] Zhang L (2019). Disrupted folate metabolism with anesthesia leads to myelination deficits mediated by epigenetic regulation of ERMN. EBioMedicine.

[20] Kostopanagiotou G (2005). Recovery and cognitive function after fentanyl or remifentanil administration for carotid endarterectomy. J. Clin. Anesth..

[21] Eckenhoff RG (2004). Inhaled anesthetic enhancement of amyloid-beta oligomerization and cytotoxicity. Anesthesiology.

[22] Johnson T (2002). Postoperative cognitive dysfunction in middle-aged patients. Anesthesiology.

[23] Ancelin ML (2001). Exposure to anaesthetic agents, cognitive functioning and depressive symptomatology in the elderly. Br. J. Psychiatry.

[24] Findeis MA (2000). Approaches to discovery and characterization of inhibitors of amyloid beta-peptide polymerization. Biochim. Biophys. Acta.

[25] Romas SN (1999). APOE genotype, plasma lipids, lipoproteins, and AD in community elderly. Neurology.

[26] Lee JE (2011). Folate intake and risk of colorectal cancer and adenoma: Modification by time. Am. J. Clin. Nutr..

[27] Scott TM (2017). B-Vitamin therapy for kidney transplant recipients lowers homocysteine and improves selective cognitive outcomes in the Randomized FAVORIT ancillary cognitive trial. J. Prev.. Alzheimers Dis..

[28] Durga J (2007). Effect of 3-year folic acid supplementation on cognitive function in older adults in the FACIT trial: A randomised, double blind, controlled trial. Lancet.

[29] Dichgans M, Leys D (2017). Vascular cognitive impairment. Circ. Res..

[30] Hogervorst E (2017). Nutrition research in cognitive impairment/dementia, with a focus on soya and folate. Proc. Nutr. Soc..

[31] Goyette P (1994). Human methylenetetrahydrofolate reductase: Isolation of cDNA, mapping and mutation identification. Nat. Genet..

[32] Chen Z (2001). Mice deficient in methylenetetrahydrofolate reductase exhibit hyperhomocysteinemia and decreased methylation capacity, with neuropathology and aortic lipid deposition. Hum. Mol. Genet..

[33] Kelly TL (2005). Infertility in 5,10-methylenetetrahydrofolate reductase (MTHFR)-deficient male mice is partially alleviated by lifetime dietary betaine supplementation. Biol. Reprod..

[34] Sahiner UM (2014). Methylene tetrahydrofolate reductase polymorphisms and homocysteine level in heart defects. Pediatr. Int..

[35] Austin RC, Lentz SR, Werstuck GH (2004). Role of hyperhomocysteinemia in endothelial dysfunction and atherothrombotic disease. Cell Death Differ..

[36] Clarke R (1998). Folate, vitamin B12, and serum total homocysteine levels in confirmed Alzheimer disease. Arch. Neurol..

[37] Seshadri S (2002). Plasma homocysteine as a risk factor for dementia and Alzheimer's disease. N. Engl. J. Med..

[38] Nebes RD (2001). Relationship of deep white matter hyperintensities and apolipoprotein E genotype to depressive symptoms in older adults without clinical depression. Am. J. Psychiatry.

