# Supplementary material for: Minor allele of rs55763075 located in MTHFR is associated with the risk of cognitive impairment after anesthesia via modulating miR-34b
Source: Sci Rep. 2021 May 27;11:11157. doi: 10.1038/s41598-021-90229-z (PMC8159953; doi:10.1038/s41598-021-90229-z)

**Minor allele of rs55763075 located in MTHFR is associated with the risk of cognitive impairment after anesthesia via modulating miR-34b**

Si-ying Li[1†], He-shou Lei[1†], Xiao-yun Wu[1], Kai Li[1], Zhi-min Liu[1], Jian-hui Lu[1], Xiao-yun Chen[1*]

1. Department of Anesthesiology, Wuming Hospital of Guangxi Medical University, Nanning 530199, P.R. China

[†]Si-ying Li and [†]He-shou Lei contributed to this work equally.

[*] Correspondence to: Xiao-yun Chen, Department of Anesthesiology, Wuming Hospital of Guangxi Medical University, No.26,Yongning Road, Wuming District, Nanning, Guangxi Zhuang Autonomous Region, P.R. China, neuromedx@yeah.net

**Running title:** Single Nucleotide Polymorphism of Methylenetetrahydrofolate reductase gene and folic acid in Cognitive Dysfunction

**Key words:** SNP, Folic acid, miRNA-34b, Cognitive impairment after anesthesia, MTHFR

**Original WB blots-uncropped:**

Fig 5B-MTHFR

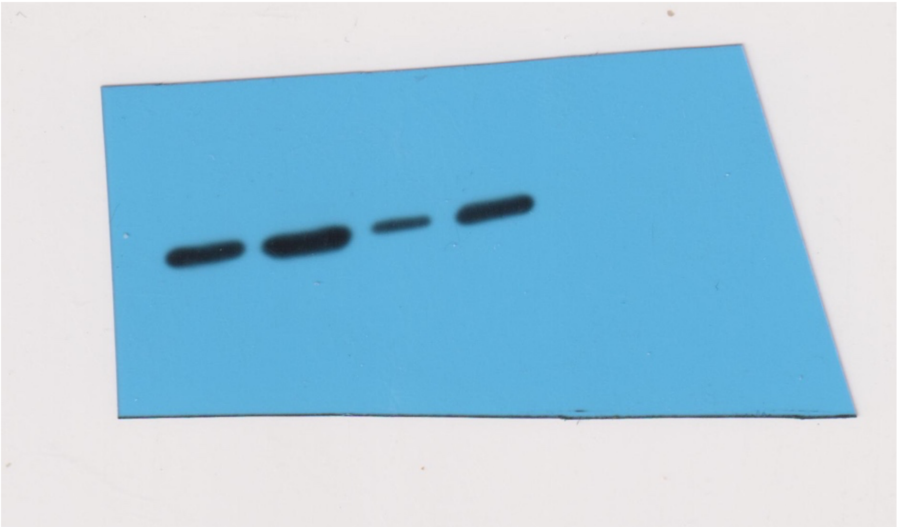

Fig 5B-beta actin

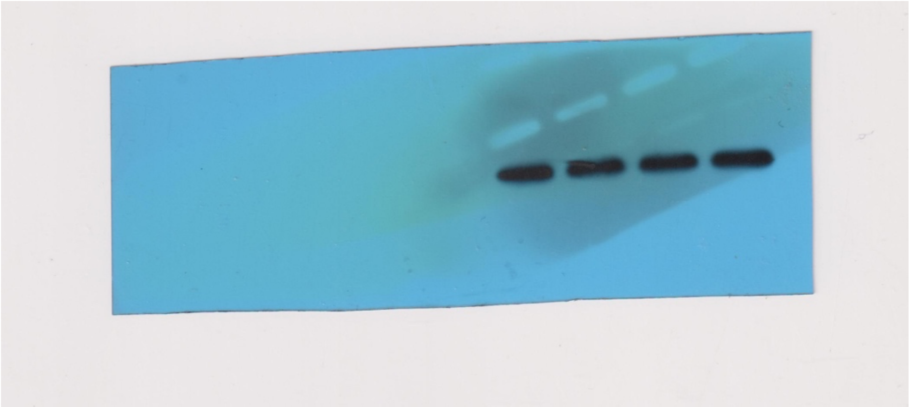

Fig 5D-MTHFR

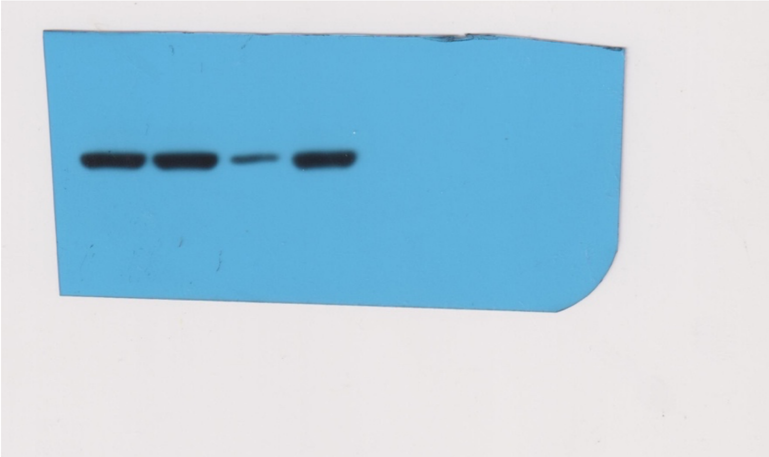

Fig 5D-beta actin

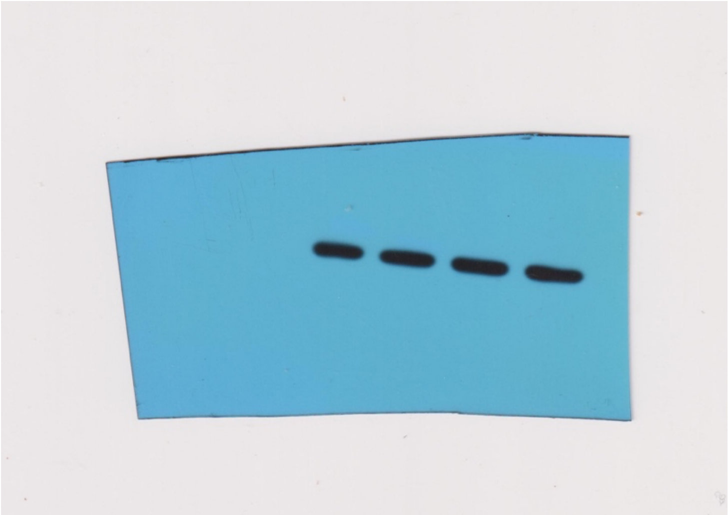

Supplement: Supplementary file 1 — Supplementary Information. [file 41598_2021_90229_MOESM1_ESM.pdf]
